# Persistent Air Embolism after Blunt Chest Trauma with Recovery to Pre-Existing Consciousness Level: A Case Report and Literature Review

**DOI:** 10.1089/neur.2021.0052

**Published:** 2022-01-20

**Authors:** Mitsuhito Soh, Toru Hifumi, Shutaro Isokawa, Tsutomu Iwasaki, Norio Otani, Shinichi Ishimatsu

**Affiliations:** Department of Emergency and Critical Care Medicine, St. Luke's International Hospital, Tokyo, Japan.

**Keywords:** blunt chest trauma, cerebral air embolism, cerebral infarction, pulmonary contusion

## Abstract

We report the case of a 71-year-old woman in whom cerebral air embolism resulted from blunt chest trauma. The woman had been lying on her left side for a while after the injury, and air traveled to the right side of the brain. As a result, a cerebral infarction occurred in the right cerebral hemisphere that caused loss of consciousness for more than 40 days. The patient recovered consciousness eventually; thus, it is important to monitor the improvement in a patient's state of consciousness, with repeated multi-modality imaging evaluations over a long period.

## Introduction

Cerebral air embolism (CAE) is caused by air bubbles in the vascular system. These bubbles obstruct the intracranial blood vessels and lead to symptoms of cerebral ischemia.^1,2^ The CAE is an iatrogenic condition associated with catheterization procedures^3–5^ or surgery^5–8^ and can result from pressure trauma from positive pressure ventilation^9–11^ or decompression sickness.^12,13^ Cerebral air often disappears during initial treatment, such as resuscitation, during imaging, and during surgery, especially in cases of trauma; in such cases, early definitive diagnosis with computed tomography (CT) is difficult.^2,14–16^

The pathogenesis and pathophysiological features of trauma-related CAE (e.g., air entry site, timing, location, and duration of CAE) have been reported^1, 2,15,17,18^; however, these mechanisms are not clearly understood. We report a characteristic case of CAE that resulted from chest trauma, and we discuss the pathogenesis and pathophysiological features of CAE.

## Case Report

A 71-year-old woman was brought to our hospital by ambulance. She had been riding in the back seat of a van that was the third vehicle in a pileup of seven passenger cars when the car in front of her backed up suddenly and hit her vehicle at a speed of 50 km/h. She was not wearing a seat belt, and her head and the right side of her chest hit the right wall of the car. She then recoiled and fell to the floor on the opposite side of the car. When the ambulance team arrived, she was still on the floor of the rear seat, lying on her left side. She was then transported to St. Luke's International Hospital.

The patient's medical history included the following: (1) cerebral infarction, which caused incomplete paralysis of the left upper and lower limbs, and (2) chronic kidney disease, for which she was being treated with dialysis. On arrival, her Glasgow Coma Scale (GCS) score was E3V5M6, pupils were 3 mm/3 mm bilaterally with no difference between the right and left sides, and the contrast reflex was normal, body temperature was 35.9°C, respiratory rate was 28 breaths/min, blood pressure was 120/81 mm Hg, heart rate was 107 beats/min, and oxygen saturation was 95% (with nasal cannula, 3 L/min).

**FIG. 1.**
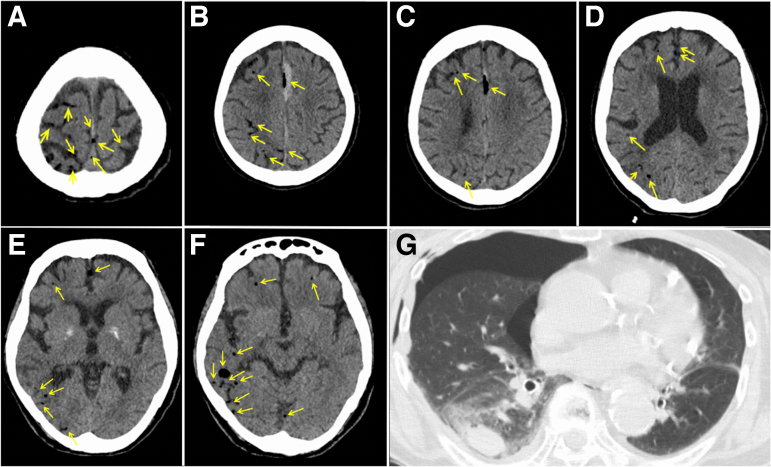
Computed tomographic images at the time of visit. (A**–F**) The air was in the right side of the brain and on the brain surface. Yellow arrows indicate air. (**G**) Right traumatic hemopneumothorax, pulmonary contusion, and multiple rib fractures were observed.

A physical examination revealed no gross trauma to the head, but respiratory depression and subcutaneous emphysema were observed in the right side of the chest. Head CT showed no fracture in the skull, but air on the right side of the brain was observed (Fig. 1A–F). In addition, an acute subdural hematoma was found on the left side of the cingulate gyrus. Chest CT showed right hemopneumothorax, pulmonary contusion, and fractures of multiple ribs (nos. 3–9) on the right side, and a chest drain was inserted (Fig. 1G).

**FIG. 2.**
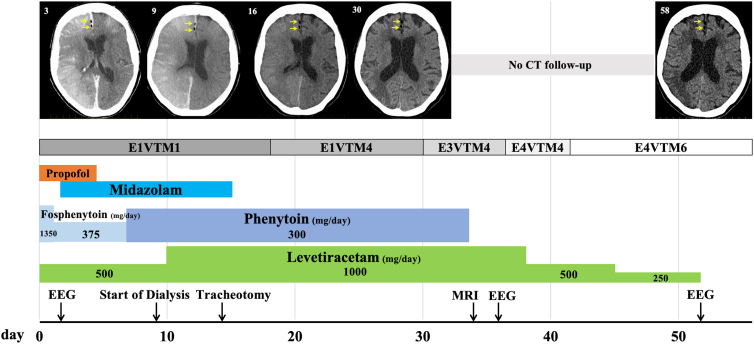
Progress on head computed tomography (CT, day 3, 9,16,30, and 58), level of consciousness (GCS), and medications for seizure control. Head CT showed prolonged air retention in the frontal lobe. Yellow arrows indicate air. MRI, magnetic resonance imaging; EEG, electroencephalography.

All these findings indicated that the air in the brain was CAE caused by chest trauma. Convulsive seizures occurred after the imaging examination, and tracheal intubation was performed while the patient was under deep sedation with continuous intravenous propofol. In addition, fosphenytoin and levetiracetam were administered as anticonvulsants (Fig. 2).

The day after admission, three-dimensional computed tomographic angiography (CTA) and computed tomographic venography (CTV) were performed; however, the results of CTA and CTV were inconclusive. The first electroencephalography (EEG) on the day after admission showed occasional spike waves in the right CPO (central, parietal, occipital), region and twitching movements in the left upper extremity. Therefore, continuous intravenous midazolam was added, and the dosage of anticonvulsants was adjusted (Fig. 2).

The cerebral edema worsened slightly on days 3 and 6, but dialysis was not performed out of concern about electrolyte imbalance. On day 9, the edematous changes in the right cerebrum became more severe, and low-efficiency dialysis was started. By day 30, although the air in the cingulate gyrus remained, the swelling of the right cerebral hemisphere had generally improved, and the cerebral sulcus was visible on imaging. At this time, the patient started to open her eyes in response to being called by her name.

**FIG. 3.**
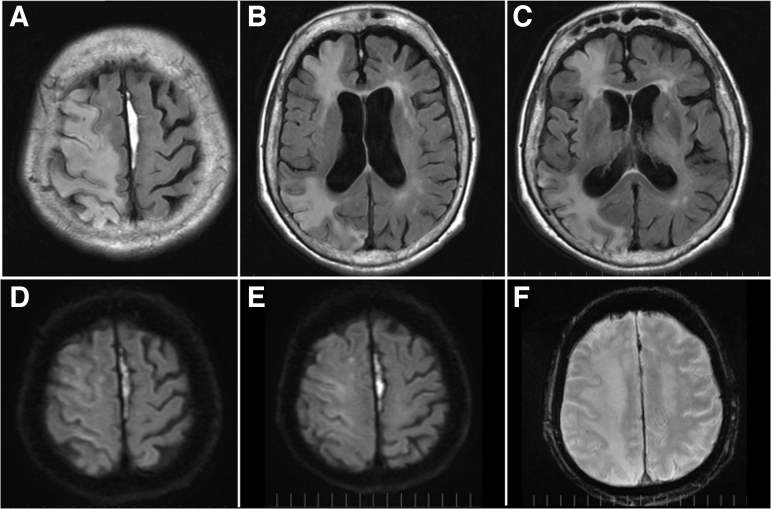
Magnetic resonance imaging on day 34. (**A–C**) Fluid-attenuated inversion recovery imaging showed a patchy high signal in the right cerebral hemisphere. (**D,E**) Diffusion-weighted images showed high signal in the right frontal cortex and center of the semicircle. (**F**) T2* (star) -weighted image showed a punctate low signal in the subcortical area of the right parietal lobe, suggestive of an old microhemorrhage.

Magnetic resonance imaging (MRI) on day 34 showed a subacute cerebral infarction with high signal along the cortex of the right cerebral hemisphere on T1-weighted image and mottled high signal in the right cerebral hemisphere on fluid-attenuated inversion recovery images (Fig. 3A–C), which was thought to be associated with CAE. Diffusion-weighted images showed high signal in the right frontal cortex and center of the semicircle (Fig. 3D, E). T2* (star) -weighted image showed a punctate low signal in the subcortical area of the right parietal lobe, suggestive of an old microhemorrhage (Fig. 3F).

An EEG (2nd time) was performed on day 36, considering the possibility of nonconvulsive status epilepticus because the condition of M4 was persistent on GCS. An EEG on day 36 showed no convulsive waves, and the anticonvulsant was reduced (Fig. 2). On day 41, the patient could follow our instructions well. The last EEG was performed on day 52, and it was confirmed that there were no seizure waves. Therefore, the anticonvulsant medication was terminated (Fig. 2), but there was no recurrence of convulsive seizures after that.

As a result of the prolonged disturbance of consciousness, the initial plan was to transfer the patient to a convalescent hospital. A head CT scan performed on day 58 showed residual air in the cingulate gyrus (Fig. 2). Because the patient's level of consciousness did not decline, she was transferred to a rehabilitation hospital, instead of a convalescent hospital, on day 73. At that time, the left upper and lower limbs exhibited more muscle weakness than did the right limbs. This was attributed to the patient's earlier paralysis in the left upper and lower limbs as the result of a previous cerebral infarction in the right cerebral hemisphere.

## Discussion

When the pulmonary parenchyma is damaged, the pulmonary veins and bronchial tubes come into contact with each other, and air enters the pulmonary veins with high pressure. The presence of air in the brain causes CAE.^1^ Pneumocephalus is usually caused directly by opening of the skull—traumatically (after skull fractures) or iatrogenically (after surgery).^19^ In our patient, air entry occurred through pulmonary injury; neither fracture of the skull nor other trauma besides pulmonary contusion was evident.

**FIG. 4.**
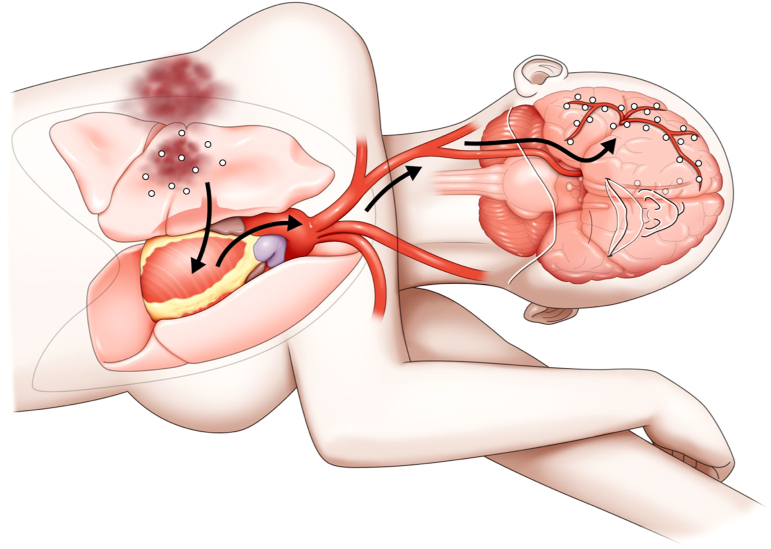
The pulmonary parenchyma was damaged, and the pulmonary veins contacted the bronchi. The air flowed from the pulmonary veins into the body circulation, and because of left lateral recumbency, the air flowed predominantly to the right side of the brain.

In this case, the distribution of air was characteristic of injury-related CAE. The distribution of air in CAE may be related to body position.^8^ After the injury, our patient was lying on her left side in the car for a long time, which may have caused the air to travel to the right side of her brain (Fig. 4). This finding was consistent with those in the literature and suggests the importance of body position at the onset of CAE.

A CAE is often difficult to diagnose definitively in the acute phase because the air shadow in the vessels of the brain on CT examination can disappear in 0.5–30 h.^7^ In trauma, in particular, the air shadow often disappears during the initial resuscitation treatment, imaging tests, surgery, and ventilator management.^8,9^ In this case, however, air continued to be detected on CT scans until day 58 of hospitalization, and it remained for a long time in the frontal lobe (Fig. 3).

The reason was thought to be the trapping of air not in the proximal arteries but near the terminal arteries of the cerebral cortex.^20^ Because the surface tension of air bubbles is inversely correlated with their diameter, smaller air bubbles are more resistant to rupture than larger bubbles. Air bubbles are thus more likely to be entrapped in small end arteries (30–60 μm in diameter)^21^ in the cortical layers than in larger proximal arteries. The flow velocity or pressure gradient is the lowest in the cortical border zone of the middle and anterior cerebral arteries, which are the most likely sites of microembolisms.^22^

It has been reported that MRI can help predict the clinical outcome of CAE.^3^ In our patient, MRI performed 34 days after the injury (Fig. 2) showed that the infarct foci in the brain were speckled, which we thought was because the air did not lead to complete occlusion. The patient had impaired consciousness for more than 40 days, but her level of consciousness improved sufficiently enough for her to speak. The causes of the prolonged disturbance of consciousness were diverse, including CAE, convulsive seizures, prolonged use of sedatives, cerebral edema, and uremic encephalopathy, and could not be pinpointed to a single cause. Because the affected part of the brain appeared narrow on MRI, neurological function recovery was thought possible.

We compared this case with those in previously published reports of CAE caused by blunt chest trauma (Table 1). In two patients, CAE was diagnosed clinically because of the absence of air on CT and the presence of mottled infarct foci on MRI; these patients had good outcomes.^15,16^ The outcome was also good in patients with air detected on CT but confined to the brain surface or with only a few small areas of air.^23,24^ In contrast, in three patients who died,^25–27^ air visible on CT was widespread. In addition, CT showed air in areas other than the brain, such as the aorta and coronary arteries. This finding supported the general theory that even a small amount of air can cause ischemia and infarction in organs^28^ and that actual symptoms and death in patients with intra-arterial gas depend on the amount and rate of air introduction.^29^

**Table 1.** Summary of Published Reports of Patients with Cerebral Air Embolism after Blunt Chest Trauma

Year	Age/sex	Chest trauma	Distribution of air in CT	Location of the cerebral infarction	Detection of air outside the brain	Outcome
2002	37/M	Bilateral pneumothorax Bilateral pulmonary contusion Bilateral multiple rib fracture	None	Bilateral cerebral hemispheres but mottled	None	Full recovery
2012	46/F	Left hemothorax, right lung contusion, right multiple rib fracture	None	Left cerebellum, left occipital lobe, left medulla oblongata but mottled	None	Full recovery
2016	28/M	Bilateral pulmonary contusion	Brain surface of bilateral cerebral hemispheres	None	Brachiocephalic artery bilateral internal carotid arteries	Full recovery
2000	20/F	Right tension pneumothorax	Several locations in the right cerebral hemisphere	Unknown (no MRI)	None	Full recovery
2001	75/M	Left lung contusion	Right cerebral hemisphere (extensive)	Unknown (no MRI)	Aorta	Dead
2008	19/M	Bilateral pulmonary contusion	Right cerebral hemisphere (extensive)	Unknown (no MRI)	Right superior pulmonary vein Left ventricle	Dead
2011	13/M	Right lung contusion, right multiple rib fracture	Bilateral cerebral hemispheres (right brain dominant)	Unknown (no MRI)	Left atrium, left ventricle Coronary artery	Dead
2021 (this case)	71/F	Right hemopneumothorax, right lung contusion, right multiple rib fracture	Right dominant, bilateral peripheral	Right cerebral hemisphere but mottled	None	Minor disability

CT, computed tomography; MRI, magnetic resonance imaging.

In our patient, the air did not reach many areas of the brain, but what remained in other areas may have affected the circulation adversely. The CT scans showed air in the right hemisphere but only on the surface of the brain, and the infarct zone on MRI was mottled and not extensive. The prognosis may have been worse if extensive air was found on CT or if air had remained in vessels other than those of the brain. In patients with severe neurological symptoms, however, if the MRI findings are minor and no residual air remains in vessels other than those in the brain, the prognosis may be good, and long-term follow-up is required.

## Conclusion

We treated a patient with CAE who had persistent air in the right frontal lobe. Air entered as a result of lung contusion immediately after the injury and traveled to the right cerebral hemisphere because the patient lay on her left side. The air was able to travel to the frontal lobe because tiny air bubbles persisted for a long time without rupture in the blood vessels.

## Abbreviations Used

CAE: cerebral air embolism
CT: computed tomography
GCS: Glasgow Coma Scale
CTA: computed tomography angiography
CTV: computed tomography venography
EEG: electroencephalography
MRI: magnetic resonance imaging

## References

[1] Ho, A.M., and Ling, E. (1999). Systemic air embolism after lung trauma. Anesthesiology 90, 564–575. 9952165 10.1097/00000542-199902000-00033

[2] Mercurio, I., Capano, D., Torre, R., Taddei, A., Trojano, G., Scialpi, M., and Gabbrielli, M. (2018). A case of fatal cerebral air embolism after blunt lung trauma: postmortem computed tomography and autopsy findings. Am. J. Forensic Med. Pathol. 39, 61–68. 29278540 10.1097/PAF.0000000000000375

[3] Chang, C., Dughi, J., Shitabata, P., Johnson, G., Coel, M., and McNamara, J.J. (1988). Air embolism and the radial arterial line. Crit. Care Med. 16, 141–143. 3342625 10.1097/00003246-198802000-00009

[4] Lowenstein, E., Little, J.W., and Lo, H.H. (1971). Prevention of cerebral embolization from flushing radial-artery cannulas. N. Engl. J. Med. 285, 1414–1415. 5121208 10.1056/NEJM197112162852506

[5] Hatling, D., Høgset, A., Guttormsen, A.B., and Müller, B. (2019). Iatrogenic cerebral gas embolism-a systematic review of case reports. Acta Anaesthesiol. Scand. 63, 154–160. 30203491 10.1111/aas.13260

[6] Hybels, R.L. (1980). Venous air embolism in head and neck surgery. Laryngoscope 90, 946–954. 7382710 10.1002/lary.1980.90.6.946

[7] Faberowski, L.W., Black, S., and Mickle, J.P. (2000). Incidence of venous air embolism during craniectomy for craniosynostosis repair. Anesthesiology 92, 20–23. 10638894 10.1097/00000542-200001000-00009

[8] Azzola, A., von Garnier, C., Chhajed, P.N., Schirp, U., and Tamm, M. (2010). Fatal cerebral air embolism following uneventful flexible bronchoscopy. Respiration 80, 569–572. 21079412 10.1159/000321849

[9] Hamilton-Farrell, M., and Bhattacharyya, A. (2004). Barotrauma. Injury 35, 359–370. 15037370 10.1016/j.injury.2003.08.020

[10] Moon, R.E. (2019). Hyperbaric treatment of air or gas embolism: current recommendations. Undersea Hyperb. Med. 46, 673–683. 31683367

[11] Thackray, N.M., Murphy, P.M., McLean, R.F., and deLacy, J.L. (1996). Venous air embolism accompanied by echocardiographic evidence of transpulmonary air passage. Crit. Care Med. 24, 359–361. 8605815 10.1097/00003246-199602000-00030

[12] Loewenherz, J.W. (1992). Pathophysiology and treatment of decompression sickness and gas embolism. J. Fla. Med. Assoc. 79, 620–624. 1431793

[13] Kizer, K.W. (1987). Dysbaric cerebral air embolism in Hawaii. Ann. Emerg. Med. 16, 535–541. 3565866 10.1016/s0196-0644(87)80679-0

[14] Takizawa, S., Tokuoka, K., Ohnuki, Y., Akiyama, K., Kobayashi, N., and Shinohara, Y. (2000). Chronological changes in cerebral air embolism that occurred during continuous drainage of infected lung bullae. Cerebrovasc. Dis. 10, 409–412. 10971028 10.1159/000016098

[15] Brownlow, H.A., and Edibam, C. (2002). Systemic air embolism after intercostal chest drain insertion and positive pressure ventilation in chest trauma. Anaesth. Intensive Care 30, 660–664. 12413269 10.1177/0310057X0203000519

[16] Kesieme, E., Feldmann, M., Welcker, K., Linder, A., and Prisadov, G. (2012). Cerebral infarct complicating traumatic pneumatocele: a rare sequela following blunt chest trauma. Thorac. Cardiovasc. Surg. 60, Suppl. 2, e16-e18. 22549758 10.1055/s-0032-1304549

[17] Thomas, A.N. (1973). Air embolism following penetrating lung injuries. J. Thorac. Cardiovasc. Surg. 66, 533–540.

[18] Ho, A.M. (1999). Is emergency thoracotomy always the most appropriate immediate intervention for systemic air embolism after lung trauma? Chest 116, 234–237. 10424531 10.1378/chest.116.1.234

[19] Allard, E., Selim, J., and Veber, B. (2019). Pneumocephalus and pneumorachis after blunt chest trauma without spinal fractures: a case report. J. Med. Case Rep. 13, 317. 31651338 10.1186/s13256-019-2208-3 PMC6813974

[20] Jeon, S.B., Kim, J.S., Lee, D.K., Kang, D.W., and Kwon SU. (2007). Clinicoradiological characteristics of cerebral air embolism. Cerebrovasc. Dis. 23, 459–462. 17435386 10.1159/000101749

[21] Muth, C.M., and Shank, E.S. (2000). Gas embolism. N. Engl. J. Med. 342, 476–482. 10675429 10.1056/NEJM200002173420706

[22] Caplan, L.R., and Hennerici, M. (1998). Impaired clearance of emboli (washout) is an important link between hypoperfusion, embolism, and ischemic stroke. Arch. Neurol. 55, 1475–1482. 9823834 10.1001/archneur.55.11.1475

[23] Reith, G., Bouillon, B., Sakka, S.G., Defosse, J.M., Gossmann, A., and Probst, C. (2016). Massive cerebral air embolism after blunt chest trauma with full neurological recovery. CJEM 18, 62–65. 25858138 10.1017/cem.2014.78

[24] Cavadore, P., Brunat, G., Perrigault, P.F., and Colson, P. (2000). Embolie gazeuse cérébrale consécutive à un pneumothorax chez une patiente en aide inspiratoire [Cerebral arterial air embolism associated with pneumothorax in a patient with pressure support ventilation]. Ann. Fr. Anesth. Reanim. 19, 249–252. 10836109 10.1016/s0750-7658(00)00215-x

[25] Sakai, I., and Nishizawa, S. (2001). Cerebral air embolism after lung contusion. Case illustration. J. Neurosurg. 95, 909. 11702887 10.3171/jns.2001.95.5.0909

[26] Milla, F., and Cahan, M. (2008). Lethal systemic air embolism in a multitrauma patient. J. Am. Coll. Surg. 206, 591. 18308232 10.1016/j.jamcollsurg.2007.05.037

[27] Brederlau, J., Muellenbach, R.M., Wunder, C., Schwemmer, U., Kredel, M., Roewer, N., Wurmb, N. (2011). Delayed systemic air embolism in a child with severe blunt chest trauma treated with high-frequency oscillatory ventilation. Can. J. Anaesth. 58, 555–559. 21432005 10.1007/s12630-011-9485-7

[28] McCarthy, C.J., Behravesh, S., Naidu, S.G., and Oklu, R. (2016). Air embolism: practical tips for prevention and treatment. J. Clin. Med. 5, 93. 10.3390/jcm5110093 PMC5126790 27809224

[29] Cunqueiro, A., and Scheinfeld, M.H. (2018). Causes of pneumocephalus and when to be concerned about it. Emerg. Radiol. 25, 331–340. 29546674 10.1007/s10140-018-1595-x

